# Systematic Review of Factors Affecting Quality of Life After Cytoreductive Surgery with Hyperthermic Intraperitoneal Chemotherapy

**DOI:** 10.1245/s10434-020-08379-9

**Published:** 2020-04-26

**Authors:** Maleen Leimkühler, Judith E. K. R. Hentzen, Patrick H. J. Hemmer, Lukas B. Been, Robert J. van Ginkel, Schelto Kruijff, Barbara L. van Leeuwen, Geertruida H. de Bock

**Affiliations:** 1 Division of Surgical Oncology, Department of Surgery, University of Groningen, University Medical Center Groningen, Groningen, The Netherlands; 2 Department of Epidemiology, University of Groningen, University Medical Center Groningen, Groningen, The Netherlands

## Abstract

**Background:**

Previous studies have shown that, overall, quality of life (QoL) decreases within the first 3–6 months after cytoreductive surgery with hyperthermic intraperitoneal chemotherapy (CRS + HIPEC), returning to baseline levels by 6–12 months. This systematic review aims to evaluate the factors affecting QoL after CRS + HIPEC within 12 months of surgery.

**Methods:**

Electronic databases were investigated searching for articles reporting QoL with validated questionnaires up to September 2019. Risk of bias was assessed with the methodological index for non-randomized studies tool. The primary outcomes were short-term (< 6 months after surgery) and medium-term (6–12 months after surgery) determinants of QoL after CRS + HIPEC. Secondary outcomes were QoL and reported symptoms over time.

**Results:**

We included 14 studies that used 12 different questionnaires. The reported data were collected prospectively or retrospectively for 1556 patients (dropout < 50% in four studies). Overall, studies showed diminished QoL within 3 months after surgery and a recovery to baseline or greater by 12 months. QoL was negatively influenced by higher age, female sex, prolonged operation time, extensive disease, residual disease, adjuvant chemotherapy, complications, stoma placement, and recurrent disease. QoL results were comparable between studies, with dropout rates above and below 50%.

**Conclusions:**

QoL returns to baseline levels within 12 months after CRS + HIPEC provided the disease does not recur, and this recovery process is influenced by several factors.

**Electronic supplementary material:**

The online version of this article (10.1245/s10434-020-08379-9) contains supplementary material, which is available to authorized users.

Peritoneal metastases are present in advanced stages of several abdominal tumors. If left untreated, they are associated with poor prognosis, high morbidity, and reduced quality of life (QoL).^1^^–^4 Peritoneal metastasis was once considered incurable and suitable for only palliative treatment.^5^^–^7 However, in carefully selected patients with limited and resectable disease and who have no distant metastases, research has shown that aggressive cytoreductive surgery (CRS) with hyperthermic intraperitoneal chemotherapy (HIPEC) may be considered a curative treatment option.^8^ In this extensive surgical procedure, all macroscopic disease is removed from the abdominal cavity, which is then perfused with heated chemotherapy agents.^9^^–^11 Since the introduction of the CRS + HIPEC approach, multiple studies have shown improved prognosis in appropriately selected patients with peritoneal metastasis from various tumors. Today, CRS + HIPEC is even regarded as the standard of care for patients with peritoneal metastasis of colorectal origin or in those with pseudomyxoma peritonei.^5^^,^^12^^,^^13^

Despite the undoubted success of CRS + HIPEC, the approach remains a high-risk treatment with a mortality of up to 8% and a morbidity of 23–66%, even in experienced centers.^9^^,^^14^^–^19 Clinicians and patients must therefore seriously weigh the potential survival benefits against the substantial risk of treatment-related morbidity, mortality, and a potentially diminished QoL and functional status. Supporting this latter consideration, two systematic reviews were recently published in which it was suggested that patients who underwent CRS + HIPEC experienced a decrease in QoL, although this eventually returned to baseline levels within 12 months of surgery.^20^^,^^21^ However, both reviews relied on limited literature searches, and only one reported a range of QoL domains.^21^ Furthermore, neither review gave sufficient consideration to the determinants of QoL after CRS + HIPEC, such as stoma placement,^22^ disease recurrence,^22^ and dropout rates. Note that dropout rates can be highly selective and lead to the most ill patients in a cohort being underrepresented. In turn, this indicates the need for a further review of the factors affecting change in QoL after CRS + HIPEC in patients with peritoneal metastasis.

The objective of this systematic review is to identify the factors affecting QoL after CRS + HIPEC in patients with peritoneal metastasis over both the short-term (within 6 months of surgery) and the medium-term (6–12 months after surgery).

## Materials and Methods

We conducted a systematic review and reported the results according to the Preferred Reporting Items for Systematic Reviews and Meta-Analyses (PRISMA) and the Meta-analysis of Observational Studies in Epidemiology (MOOSE) guidelines.^23^^,^^24^

### Literature Search Strategy

The search strategy was developed in collaboration with an experienced medical research librarian, and a full description of the strategy can be found in Supplementary Table 1. The systematic literature search was conducted in October 2018 and updated in September 2019, using the databases of PubMed, Embase, Cochrane library (trials), and Web of Science. We aimed to identify prospective and retrospective observational studies and randomized clinical trials (RCTs) that met predefined eligibility criteria. The Medical Subject Headings (MeSH) term “Quality of Life” and different terms for “HIPEC” were used. Only original peer-reviewed research was included. No further restrictions were placed on the study design, language, or study date. Finally, the references of included articles and related review articles were manually screened to identify additional relevant studies.

### Eligibility Criteria

Studies were included for review based on the following eligibility criteria: (1) patients were treated with CRS + HIPEC, (2) reported QoL data were obtained by validated questionnaires, (3) patients with colorectal peritoneal metastasis were included, (4) data collection was prospective, and (5) research was original and peer-reviewed. To guarantee that the publications were understood at an academic level, the articles were required to be written in English, Dutch, German, or Spanish. We excluded any studies in which patients were retreated with HIPEC or another intraperitoneal chemotherapy (e.g., postoperative intraperitoneal chemotherapy). Studies reporting data only about patients with pseudomyxoma peritonei or a primary malignancy of the appendix or stomach were also excluded because of the different prognosis compared with other CRS + HIPEC indications. Finally, studies with a cross-sectional design were excluded because one measurement point is insufficient to measure the change in QoL.

### Study Selection

Titles and abstracts were independently reviewed for eligibility according to predefined criteria by two authors (M.L. and J.H.). The reviewers were not blinded to publication date, journal, or authors. The full texts of potentially eligible articles were retrieved and assessed for inclusion independently by each author. Disagreement about study inclusion was resolved by consensus or by discussion with a third author (B.L.).

### Data Extraction and Quality Assessment

Data extraction for predetermined items was performed independently by two authors (M.L. and J.H.). The following data were extracted: first author, publication year, country of origin, study years, study design, number of patients, inclusion and exclusion criteria, age, sex, tumor origin, morbidity and mortality related to CRS + HIPEC, QoL instruments used, measurement points, questionnaire response rate, and mean overall and subscale QoL scores (e.g., physical health, social health, emotional health, functional health, and cognitive health). If data were not reported, items were recorded as “NR” (not reported).

To evaluate the quality of the included articles, two reviewers (M.L. and J.H.) independently conducted a risk of bias analysis using the methodological index for non-randomized studies (MINORS) for individual studies.^25^ In the event of disagreement, consensus was reached through discussion or by consulting a third author (B.L.). The MINORS criteria were specifically developed for use with studies that have a surgical intervention. Each item can be scored as 0 (not reported), 1 (reported but inadequate), or 2 (adequately reported), resulting in global ideal scores of 16 for noncomparative studies and 24 for comparative studies. Although the tool gives an indication of the quality of studies in different domains, it has no defined cut-off scores for what constitutes high or low quality.

The interobserver reliability of the risk of bias assessment was calculated using the intraclass correlation coefficient (ICC). The ICC for interobserver reliability was interpreted according to the definition of Landis and Koch^26^ as follows: poor if < 0.00, slight if 0.00–0.20, fair if 0.21–0.40, moderate if 0.41–0.60, substantial if 0.61–0.80, and almost perfect if 0.81–1.00.

### Outcomes

The primary outcomes were the short- (< 6 months) and medium-term (6–12 months) factors affecting QoL in patients with peritoneal metastasis after CRS + HIPEC. The secondary outcome was the QoL after CRS + HIPEC in various domains after CRS + HIPEC, including overall health, physical health, emotional health, social health, functional health, and cognitive functioning, and symptoms.

## Results

### Study Selection

**Fig. 1:**
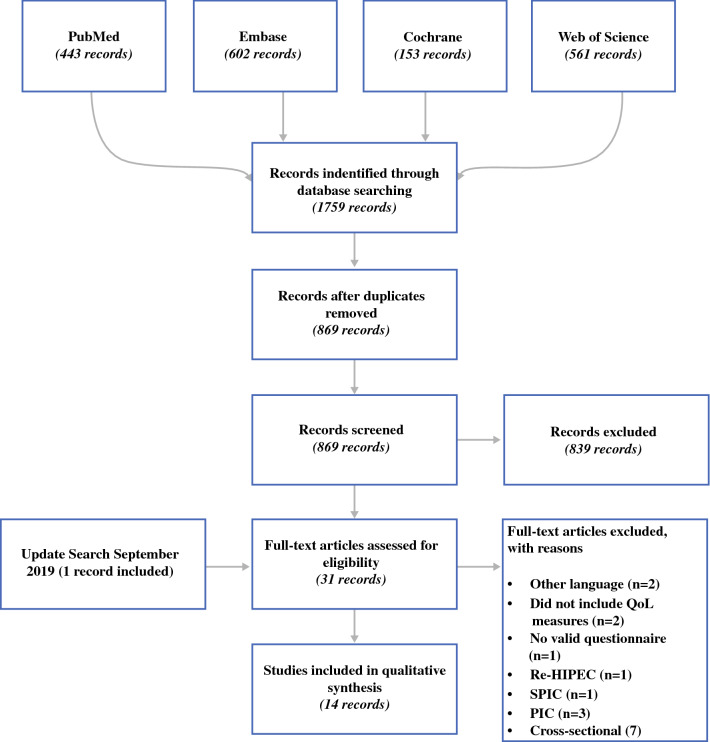
Flowchart of systematic review. *HIPEC* hyperthermic intraperitoneal chemotherapy, *IPC* intraperitoneal chemotherapy, *QoL* quality of life, *SPIC* sequential perioperative intraperitoneal chemotherapy

A total of 1759 potentially relevant records were identified from four databases (Fig. 1). After removing duplicates, titles and abstracts of the remaining 869 records were screened. This resulted in 31 full-text articles being eligible for inclusion in our review. We then excluded 17 articles based on the eligibility criteria, leaving 14 articles that met all eligibility criteria for the systematic review.

### Study Characteristics

QoL data were included for 1556 patients who had undergone CRS + HIPEC for peritoneal metastases due to a range of primary tumors (Table 1).^22^^,^^27^^–^39 There were 12 prospective and 2 retrospective studies; 8 were conducted in North America, 5 in Europe, and 1 in Asia. The studies used 12 different types of validated questionnaire to assess QoL after CRS + HIPEC. Most common among these were the Functional Assessment of Cancer Therapy (FACT), the European Organization for Research and Treatment of Cancer (EORTC) QLQ-C30, the Medical Outcomes Study Health Survey Short Form (SF-36), and the Eastern Cooperative Oncology Group (ECOG) performance status (Supplementary Table 2). Most prospective studies conducted measurements at baseline and at 3, 6, and 12 months after surgery. Characteristics of the CRS + HIPEC procedures performed in the different studies can be found in Supplementary Table 3.

**Table 1:** Main characteristics of included studies assessing QoL after CRS with HIPEC

Author/year	Country	Study period	Study design	Patients (*n*)	Control group	Primary tumor origin	QoL instrument (s)	Time points of assessment
Albertsmeier^27^	Germany	NR	P	40	No	CRC *18%*, gastric *15%*, PMP *13%*, ovarian 13%, meso *3%*	EORTC QLQ-C30	Baseline and 3, 9, and 18 months after surgery
Bayat^39^	Canada	2011 to 2017	P	158	No	Low-grade appendix *45%*, CRC, high-grade appendix, small bowel *44%*, peritoneal mesothelioma *11%*	EORTC QLQ-C30, EORTC QLQ-CR29	3, 6, and 12 months
Chia^22^	Singapore	2012 to 2015	P	23	No	CRC *100%*	EORTC QLQ-C30, EORTC QLQ-CR29	Baseline and 3, 6, and 12 months after surgery
Dodson^28^	USA	2000 to 2015	P	598	No	CRC *22%*, ovarian *3.7%*, meso *8.4%*, appendix *58%*, small bowel *2%*, others *6.2%*	SF-36, FACT-C, BPI, CES-D, ECOG	Baseline and 3, 6, 12, and 24 months after surgery
Hamilton^29^	Canada	2011 to 2014	R	42	Yes	CRC *38%*, meso *4.8%*, appendix *55%*, small bowel *2%*	EORTC QLQ-C30	6 months after surgery
Hill^30^	USA	2001 to 2009	P	61	No	CRC *100%*	SF-36, FACT-C, BPI, CES-D, ECOG	Baseline and 3, 6, and 12 months after surgery
Hinkle^31^	USA	NR	R	36	Yes	CRC *14%*, ovarian *22%*, meso *3%*, appendix *39%*, small bowel *3%*, desmoplastic small round cell tumor *11%*, primary peritoneal *6%*, fibrolamellar hepatocellular carcinoma *3%*	FACT-C	Baseline and 2 weeks, 1, 3, 6, and 12 months after surgery
Kopanakis^32^	Greece	2011 to 2015	P	80	No	CRC *24%*, gastric *6%*, PMP *19%*, ovarian *35%*, meso *9%*, endometrial *5%*, sarcoma *3%*	FACT-C	Baseline and 1, 3, 6, 12, 18 and 24 months after surgery
Macrí^33^	Italy	2003 to 2007	P	17	No	CRC *41%*, gastric *29%*, ovarian *29%*	FACT	Baseline and 3 and 6 months after surgery
McQuellon^35^	USA	1995 to 1997	P	64	No	CRC *25%*, gastric *17%*, PMP *2%*, ovarian *6%*, meso *9%*, appendix *23%*, small bowel *2%*, sarcoma *5%*, other gastrointestinal *2%*, pancreas *2%*, malignant neoplasms *8%*	FACT-C, BPI, CES-D, ECOG, ADL subscale items	Baseline and 2 weeks, 3, 6, and 9 months after surgery
McQuellon^34^	USA	1998 to 2005	P	96	No	CRC *25%*, gastric *4%*, ovarian *5%*, meso *9%*, appendix *38%*, miscellaneous *19%*	SF-36, FACT-C, BPI, CES-D, ECOG	Baseline and 3, 6, and 12 months after surgery
Passot^36^	France	2007 to 2011	P	216	No	CRC *26%*, gastric *6%*, PMP *19%*, meso *8%*, ovarian *35%*, primary serous peritoneal carcinoma *4%*, others *5%*	GIQLI	Baseline and 1, 3, 6, and 12 months after surgery
Tsilimparis^37^	Germany	2005 to 2010	P	90	No	CRC *21%*, gastric *10%*, PMP *16%*, ovarian *19%*, meso *13%*, appendix *16%*, small bowel *1%*, malignant mixed mesodermal tumor *1%*	EORTC QLQ-30	Baseline and 1, 6, 12, 24, and 36 months after surgery
Tuttle^38^	USA	2001 to 2005	P	35	No	CRC *20%*, gastric *6%*, meso *9%*, appendix *54%*, small bowel *6%*, gallbladder *3%*, unknown *3%*	FACT-C	Baseline and 4, 8, and 12 months after surgery

*ADL* subscale items, activities of daily living (10-item activities of daily living subscale is part of SF-36), *BPI* brief pain inventory, *CES*-*D* center of epidemiologic studies depression, *CRC* colorectal carcinoma, *CRS* cytoreductive surgery, *ECOG* Eastern Cooperative Oncology Group Performance status, *EORTC QLQ*-*C30/CR29* European Organization for the Research and Treatment of Cancer Core Quality of Life Questionnaires, *FACT*-*(C)* Functional Assessment of Cancer Therapy (+ colon subscale), *GIQLI* Gastro-Intestinal Quality of Life Index, *HIPEC* hyperthermic intraperitoneal chemotherapy, *Meso* peritoneal mesothelioma, *NR* not reported, *P* prospective study design, *PMP* pseudomyxoma peritonei, *QoL* quality of life, *R* retrospective study design, *SF*-*36* medical outcomes study 36-item short-form health survey

**Fig. 2:**
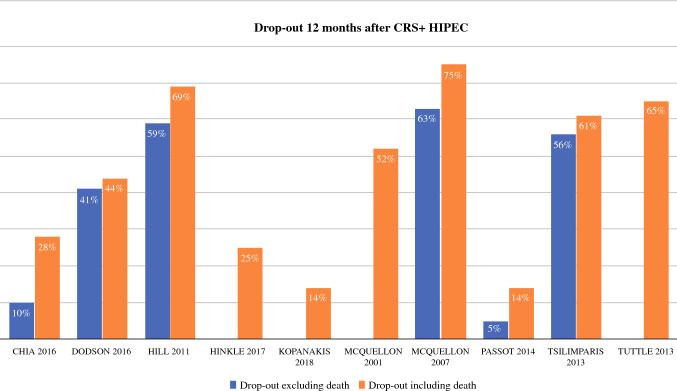
Dropout rates. *CRS* cytoreductive surgery, *HIPEC* hyperthermic intraperitoneal chemotherapy

Excluding three studies,^29^^,^^31^^,^^33^ dropout rates were 9–51% at 6 months and 10–75% at 12 months (Fig. 2)^22^^,^^28^^,^^30^^–^32^,^^34^^–^38 and were mostly explained by the high mortality of 3–18%.^22^^,^^28^^,^^30^^,^^34^^,^^36^^,^^37^ Other reasons for dropout were mentioned in one study, including that patients were too sick (13%) or refused (6%) to participate further, but with no reason recorded in many cases (32%).^30^

### Quality and Risk of Bias Assessment

To evaluate the quality of the included articles, we performed a risk of bias assessment using the MINORS criteria, the results of which are summarized in Supplementary Table 4.^25^ All of the included studies were observational, and none reached the global ideal scores. The mean MINORS score was 9.83 (range 6–11) for the noncomparative studies and 14.50 (range 14–15) for the comparative studies. None of the included studies reached the maximum scores because their aims were unclear or because we could not determine whether prospective data collection was performed according to a previously reported protocol. However, 12 studies reported their endpoints in an adequate manner. Loss to follow-up was not reported in 3 studies (16%), but it was reported in the other 11 studies (84%), where it exceeded the 5% limit applied by the MINORS criteria. Note that loss to follow-up > 5% is common in QoL research. We therefore evaluated the quality of the studies as mediocre. The ICC between the two reviewers, 0.95, was almost perfect.

### Development of QoL During Follow-Up

**Fig. 3:**
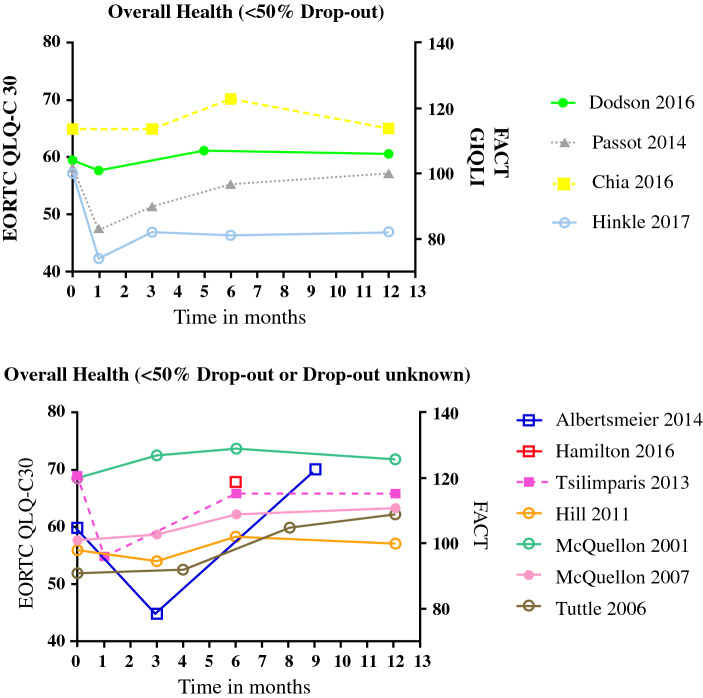
Overall QoL. *EORTC QLQ*-*C30* European Organisation for Research and Treatment of Cancer Quality of Life of Cancer Patients, *FACT* Functional Assessment of Cancer Therapy, *GIQLI* Gastrointestinal Quality of Life index, *QoL* quality of life

**Fig. 4:**
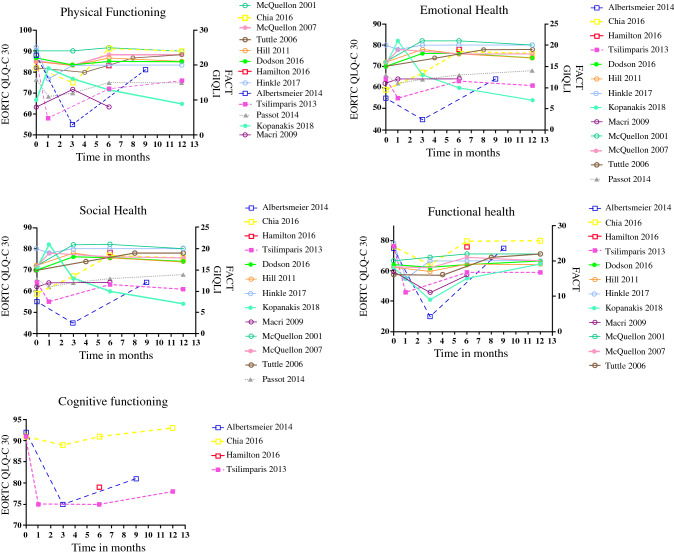
QoL by domain. *EORTC QLQ*-*C30* European Organisation for Research and Treatment of Cancer Quality of Life of Cancer Patients, *FACT* Functional Assessment of Cancer Therapy, *GIQLI* Gastrointestinal Quality of Life index, *QoL* quality of life

The general picture after CRS + HIPEC was for QoL to decrease over the first 3 months, to begin to recover by 6 months, and to reach or exceed the baseline measurements by 12 months (Fig. 3).^22^^,^^27^^–^31^,^^34^^–^38 This change in QoL was also evident in the different QoL domains, including physical health,^22^^,^^27^^–^38 emotional health,^22^^,^^27^^–^38 social health,^22^^,^^27^^–^34^,^^36^^–^38 functional health,^22^^,^^27^^–^35^,^^37^^,^^38^ and cognitive health (Fig. 4).^22^^,^^27^^,^^29^^,^^37^ Symptoms that most frequently arose or worsened within the first 6 months after surgery were fatigue, dyspnea, insomnia, and diarrhea. However, all symptoms except for diarrhea had improved by 12 months after surgery (Table 2).^22^^,^^27^^,^^28^^,^^30^^,^^34^^,^^35^^,^^37^ Several studies described an increased level of pain 3 months after CRS + HIPEC compared with baseline levels. Thereafter pain levels decreased, reaching levels lower than baseline at 6 months.^28^^,^^30^^,^^34^^,^^35^^,^^37^

**Table 2:** Overall trend of impact of CRS with HIPEC on different symptoms short- and long-term

Short-term symptom	Long-term symptom
< 6 months	6–12 months
Diarrhea**^22^^,^^27^^,^^37^	Diarrhea*^22^^,^^27^^,^^37^
Dyspnea**^22^^,^^27^^,^^37^	
Fatigue**^22^^,^^27^^,^^37^	Appetite loss***^22^^,^^27^^,^^37^
Insomnia**^22^^,^^27^	Constipation***^22^^,^^27^^,^^37^
	Depression***^28^^,^^30^^,^^34^^,^^35^
Appetite loss*^22^^,^^27^^,^^37^	Nausea and vomiting***^22^^,^^27^^,^^37^
Nausea and vomiting*^22^^,^^27^^,^^37^	
	Dyspnea^†^^22^^,^^27^^,^^37^
Depression***^28^^,^^30^^,^^34^^,^^35^	Insomnia^†^^22^^,^^27^
Constipation^‡^^22^^,^^27^^,^^37^	Fatigue^‡^^22^^,^^27^^,^^37^
Pain^‡^^22^^,^^27^^,^^28^^,^^30^^,^^34^^,^^35^^,^^37^	Pain^‡^^22^^,^^27^^,^^28^^,^^30^^,^^34^^,^^35^^,^^37^

*CRS* cytoreductive surgery, *HIPEC* hyperthermic intraperitoneal chemotherapy

*Nonsignificant increase of symptom compared with prior assessment (e.g., preoperative assessment or within 6 months after surgery assessment)

**Significant increase of symptom compared with preoperative assessment (*p* < 0.005)

***Nonsignificant decrease of symptom compared with prior assessment

^†^Significant decrease of symptom compared with prior assessment

^‡^Increase of symptom above preoperative assessment

### Determinants of QoL

A variety of patient-, tumor-, and treatment-related factors were evaluated to determine their impact on QoL after CRS + HIPEC (Table 3).^22^^,^^27^^,^^29^^,^ 30^,^^32^^,^^33^^,^ 35^,^^36^^,^^38^^,^^39^ Factors that negatively influenced QoL after CRS + HIPEC were higher age,^32^^,^^33^ female sex,^27^^,^^32^ prolonged operation time,^22^^,^^36^ high completeness of cytoreduction (CC) score,^22^^,^^32^^,^^33^^,^ 36 treatment with adjuvant chemotherapy,^22^^,^^33^ postoperative complications,^29^^,^^38^ presence of a stoma,^22^^,^^32^^,^^36^ and disease recurrence within 12 months.^22^^,^^36^ A high Peritoneal Cancer Index (PCI) before CRS + HIPEC also influenced the QoL negatively,^22^^,^^32^^,^^36^ although one study found a statistically nonsignificant negative effect of the PCI on QoL in the first 6 months and a positive effect in the subsequent 6 months.^27^

**Table 3:** Determinants of QoL after CRS with HIPEC

Determinant	Result	References
Age (years)	Younger age leads to a quicker recovery of QoL	Kopanakis^32^^,^Macrí 33
Sex	Female sex is associated with lower QoL and lower emotional health	Albertsmeier^27^, Kopanakis^32^
Primary tumor site	Ovarian carcinomas show slower recover of QoL Colon and gastric cancer show lower QoL but higher emotional health	Albertsmeier^27^, Macrí^33^
Malignant ascites	Patients with malignant ascites show lower baseline scores of QoL but, after operation, immediate increase of QoL Patients without malignant ascites experience a decrease of QoL at 3 months, after which recovery begins	McQuellon^35^
Prolonged operation time	Longer duration of surgery is associated with diminished QoL	Chia^22^^,^Passot^36^
PCI	Patients with higher PCI suffer from greater decrease in QoL One study did not find a difference in QoL in patients with high or low PCI	Chia^22^^,^Kopanakis^32^^,^Passot^36^^,^Albertsmeier^27^
CC score	Patients with higher CC score experience higher decrease of QoL	Chia^22^^,^Kopanakis^32^^,^Macrí^33^^,^Passot^36^
Type of bowel resection	Type of bowel resection does not influence QoL Social function scores are lower in patients with lower anterior resection	Bayat^39^
Adjuvant chemotherapy	Patients who receive adjuvant chemotherapy show lower QoL after CRS + HIPEC	Macrí^33^^,^Chia^22^
Complication	Patients who experienced complications show slower recovery of QoL	Tuttle^38^^,^Hamilton^29^
Presence of Stoma	Presence of a stoma is negatively associated with QoL	Chia^22^^,^Kopanaki^32^^,^Passot^36^
Recurrence	Recurrence within 1 year leads to a lower QoL	Chia^22^^,^Passot^36^

*CC score* Completeness of Cytoreduction Score, *CRS* cytoreductive surgery, *HIPEC* hyperthermic intraperitoneal chemotherapy, *PCI* Peritoneal Cancer Index, *QoL* quality of life

When comparing studies by dropout rate, there was no difference in overall QoL between the studies with high (> 50%) and low (< 50%) rates (Fig. 3). Results were comparable for each domain (data not presented).

## Discussion

This systematic review identified several factors that negatively influence QoL after CRS + HIPEC. These factors are higher patient age, female sex, prolonged operation time, extensive disease (higher PCI), more residual disease after surgery (higher CC score), adjuvant chemotherapy, postoperative complications, stoma placement, and disease recurrence. It was striking that dropout rates did not affect these results. Overall, most patients experienced a significant decline in a broad range of QoL domains during the first months after CRS + HIPEC, but generally recovered to preoperative levels by 6–12 months after surgery.

The recovery process over the first year after CRS + HIPEC appears to be promising, but these results should be interpreted with caution. Although we found no difference in QoL among studies based on their dropout rates, we must remember that our results only apply to patients who remained in the studies. Only 4 of 14 studies reported QoL data based on over half of their enrolled populations, and patient deaths only explained a small amount of the dropouts. Most dropouts were for other reasons, such as patients being too weak to continue with the study due to either disease recurrence or significant symptomatology.^30^ Consequently, QoL may be overestimated after CRS + HIPEC. However, only one study reported on the reasons for dropout, so it is difficult to evaluate the possible overestimation of QoL.

Another relevant issue was that the included studies were of mediocre quality based on the MINORS criteria. This included having small sample sizes (only five studies included more than 80 patients) and heterogeneous patient groups with a variety of primary tumors. The reported QoL of patients might be more determined by the different tumor types and stages than the other factors. To evaluate the impact of tumor type and stage on QoL, studies are needed that stratify their data by tumor type and stage. Moreover, some important factors that could have affected QoL (e.g., patient characteristics, tumor characteristics, HIPEC regimens, and postoperative morbidity) were only analyzed in a few studies.^22^^,^^31^^,^^33^ It was also notable that there had been no consideration as to whether some factors were associated with each other.

A strength of the included studies was that they all used standardized and validated questionnaires. However, the results may fail to give a comprehensive view of the QoL of patients after CRS + HIPEC specifically, not least because the applied questionnaires were not designed for this purpose. Furthermore, the studies used different validated questionnaires to assess QoL and measured QoL at differing times. In studies using in-depth semi structured interviews to collect QoL data after CRS + HIPEC, physical symptoms were reported to persist in at least half of the patients between 6 and 12 months after surgery. These included chronic pain, diet restrictions, ongoing gastrointestinal problems, and sleep difficulties.^40^ In other cases, patients described crying spells, depression, and stoma-related problems (e.g., social issues, negative effect on intimate relationships, and constant reminder of disease),^41^ as well as uncertainty about the future or death.^42^ These symptoms and their impact on QoL can be missed when using questionnaires that are not disease specific. This may be rectified by developing a standardized and validated questionnaire for use after CRS + HIPEC.

Most included studies described that QoL domains returned to baseline levels after CRS + HIPEC. However, it must be remembered that baseline QoL levels were measured shortly before the operation, when patients might have already been suffering from clinical symptoms of their disease. Therefore, QoL may have been already lower compared with the QoL before the onset of the disease, as it has been reported in patients suffering from malignant ascites before CRS + HIPEC.^35^ Therefore, it is questionable whether we should consider a return to baseline QoL a sufficient metric. It has been shown that patients who received CRS + HIPEC and remained disease-free during follow-up scored higher on overall health than patients who developed untreatable recurrent or metastatic disease.^43^ After 1 year of follow-up, their QoL scores were also reported as comparable to those of patients with cancer who undergo surgery without HIPEC and to those of patients who are disease free and functioning well.^44^^,^^45^ However, QoL scores are still reported to be lower than in the general population.^46^ Clinicians and patients must therefore consider that QoL may remain lower than before the disease first developed. When making clinical decisions, we advocate that practitioners consider the patient’s expectations and their perspectives regarding QoL, life goals, and other influential factors.

In conclusion, this review shows that QoL after CRS + HIPEC tends to be negatively affected by certain patient characteristics, procedure-specific outcomes, and postoperative disease. Notably, study dropout rates did not affect these factors. Although most patients experience a significant decline in a broad range of QoL domains during the first few months after CRS + HIPEC, they generally recover to preoperative levels by 6–12 months. Future research should now focus on study designs that can describe the profound experiences of patients who have undergone CRS + HIPEC. Given that factors affecting QoL can only be partially influenced, it is essential that patients receive detailed and honest counseling about these outcomes before CRS + HIPEC.

## Electronic supplementary material

Below is the link to the electronic supplementary material.

Supplementary material 1 (DOCX 15 kb)

Supplementary material 2 (DOCX 22 kb)

Supplementary material 3 (DOCX 24 kb)

Supplementary material 4 (DOCX 20 kb)
