# Supplementary material for: Systematic Review of Factors Affecting Quality of Life After Cytoreductive Surgery with Hyperthermic Intraperitoneal Chemotherapy
Source: Ann Surg Oncol. 2020 Apr 26;27(10):3973–83. doi: 10.1245/s10434-020-08379-9 (PMC7471142; doi:10.1245/s10434-020-08379-9)
Supplement: Supplementary file 1 — Supplementary material 1 (DOCX 15 kb) [file 10434_2020_8379_MOESM1_ESM.docx]

**Supplementary Table 1. Search algorithm for the systematic review**

| **Pubmed (443 records) *** |
| --- |
| (HIPEC[tiab] OR hypertherm*[tiab] OR ((intra-peritoneal[tiab] OR intraperitoneal[tiab]) AND chemotherap*[tiab])) AND ("Quality of Life"[Mesh] OR Life Quality[tiab] OR quality of life[tiab] OR QoL[tiab] OR HRQoL[tiab]) |
| **Embase (602 records) *** |
| ('hyperthermic intraperitoneal chemotherapy'/exp OR (HIPEC OR hypertherm* OR ((intra-peritoneal OR intraperitoneal) AND chemotherap*)):ab,ti) AND ('quality of life'/exp OR (‘Life Quality’ OR ‘quality of life’ OR QoL OR HRQoL):ab,ti) NOT 'conference abstract'/it |
| **Cochrane library (153 records) *** |
| (HIPEC OR hypertherm* OR ((intra-peritoneal OR intraperitoneal) AND chemotherap*)) AND (“quality of life” OR “life quality” OR QoL OR HRQoL) |
| **Web of Science (561 records) *** |
| TS=(HIPEC OR hypertherm* OR ((intra-peritoneal OR intraperitoneal) AND chemotherap*)) AND TS=(“quality of life” OR “life quality” OR QoL OR HRQoL) |

***** Research performed on October 24, 2018.
