# Supplementary material for: Systematic Review of Factors Affecting Quality of Life After Cytoreductive Surgery with Hyperthermic Intraperitoneal Chemotherapy
Source: Ann Surg Oncol. 2020 Apr 26;27(10):3973–83. doi: 10.1245/s10434-020-08379-9 (PMC7471142; doi:10.1245/s10434-020-08379-9)
Supplement: Supplementary file 2 — Supplementary material 2 (DOCX 22 kb) [file 10434_2020_8379_MOESM2_ESM.docx]

**Supplementary Table 2. Outline of the different QoL assessment tools used in the included studies that assessed QoL after CRS with HIPEC**

| **QoL instruments** | **Description** | **Different domains** *(scoring range)* |
| --- | --- | --- |
| **ADL subscale items** | - 10-items self-report questionnaire - ADL subscale items are a part of the SF-36 QoL scale | - *Effect of pain on ADL* (0-100) |
|  |  |  |
| **BPI** | - 14-items self-report questionnaire assessing pain severity, location, and impact on function the past seven days | - *Intensity of the pain* (0-40) - *Interference of pain* (0-70) |
|  |  |  |
| **CES-D** | - 20-items self-report questionnaire to detect depressive orders (according to DSM-IV criteria) | - *Depressive symptoms* (0-60) |
|  |  |  |
| **ECOG** | - Single-item self-report questionnaire | - *Performance status* (0-4) |
|  |  |  |
| **EORTC**  **QLQ-CR29** | - Tumor-specific, 29-items self-report questionnaire for colorectal cancer patients | - *Functional scales* (0-100): body image, anxiety, weight, and sexual interest. - *Symptom scales* (0-100) |
|  |  |  |
| **EORTC**  **QLQ-C30** | - 30-items self-report questionnaire - EORTC QLQ-C30 is intended to be supplemented by different tumor-specific questionnaire modules. | - *GHS* (0-100) - *Functional scales* (0-100): physical functioning, role functioning, emotional functioning, cognitive functioning, and social functioning. - *Symptom scales* (0-100) |
|  |  |  |
|  |  |  |
| **FACT-C** | - Tumor-specific, 36-items self-report questionnaire for patients with colorectal PM | - *Scales according to FACT-G* - *9-item colon subscale (CCS)* |
|  |  |  |
|  |  |  |
| **GIQLI** | - 36-items self-report questionnaire | - *Functional scales* (0-64): physical function, feelings, and social integration - *Symptom scale* (0-76) - *Effect of medical treatments* (0-4) |
|  |  |  |
|  |  |  |
| **SF-36** | - 36-items self-report questionnaire | - *GHS* (0-100) - *Functional scales* (0-100): physical functioning (PF), role physical (RP), role emotional (RE), bodily pain (BP), vitality (VT), social functioning (SF), and mental health (MH) - *Mental component summary* (MCS) - *Physical component summary* (PCS) |

Abbreviations: ADL subscale items, activities of daily living – 10-item activities of daily living subscale is part of SF-36; BP, bodily pain; BPI, brief pain inventory; CCS, 9-item colon subscale; CES-D, center of epidemiologic studies depression; CRS cytoreductive surgery; DSM Diagnostic and Statistical Manual of Mental Disorder; ECOG eastern cooperative oncology group performance status; EORTC QLQ-C30/CR29, European organization for the research and treatment of cancer core quality of life questionnaires; EWB emotional well-being; FACT-(C) functional assessment of cancer therapy (+ colon subscale); FWB, functional well-being; GIQLI, gastrointestinal quality of life index; GHS, global health status; HIPEC, hyperthermic intraperitoneal chemotherapy; MH, mental health; MCS, mental component summary; PCS, physical component summary; PF, physical functioning; PM, peritoneal metastases; PWB, physical well-being; QoL, quality of life; RE, role emotional; RP, role physical functioning; SF, social functioning; SF-36, medical outcomes study 36-item short-form Health survey; SWB, social well-being; VT, vitality.
