# Supplementary material for: Systematic Review of Factors Affecting Quality of Life After Cytoreductive Surgery with Hyperthermic Intraperitoneal Chemotherapy
Source: Ann Surg Oncol. 2020 Apr 26;27(10):3973–83. doi: 10.1245/s10434-020-08379-9 (PMC7471142; doi:10.1245/s10434-020-08379-9)
Supplement: Supplementary file 3 — Supplementary material 3 (DOCX 24 kb) [file 10434_2020_8379_MOESM3_ESM.docx]

**Supplementary Table 3. Treatment characteristics and postoperative outcomes in the studies of QoL after CRS with HIPEC**

| **Author/year** | **PCI at HIPEC, median (range)** | **Chemotherapeutic agent(s)** | **HIPEC**  **technique** | **Perfusion**  **period, min** | **Perfusion temperature, °C** | **Overall morbidity,**  **n (%)** | **Severe morbidity,**  **n (%)^₪^** | **Mortality, n (%)^¥^** |
| --- | --- | --- | --- | --- | --- | --- | --- | --- |
| **Albertsmeier 2014** | NR | MMC, Cisplatin, Oxaliplatin, Docetaxel | Open | 60 | 41 | 14 *(35)* | NR | **30-day mortality:** NR  **Overall mortality:** 7 *(22)* |
|  |  |  |  |  |  |  |  |  |
| **Chia 2016** | 8 *(0-27)* | MMC | Closed | 60 | 42 | 6 *(24)* | 3 *(12)* | **30-day mortality:** NR  **Overall mortality:** 3 *(13)* |
|  |  |  |  |  |  |  |  |  |
| **Dodson 2016** | 14 *(NR)** | MMC, Cisplatin, Oxaliplatin, Carboplatin, Mitoxantrone | Closed | 120 | 40 | 406 *(68)* | 130 *(22)* | **30-day mortality:** 21 *(4)*  **Overall mortality:** NR |
|  |  |  |  |  |  |  |  |  |
| **Hamilton 2016** | 16 *(3-39)* | MMC, Oxaliplatin, 5-FU, leucovorin | Closed | 30 or 90 | NR | NR | 14 *(33)* | NR |
|  |  |  |  |  |  |  |  |  |
| **Hill 2011** | NR | MMC | Closed | 90 | 40 | 29 *(48)* | NR | **30-day mortality:** NR  **Overall mortality:** 15 *(25)* |
|  |  |  |  |  |  |  |  |  |
| **Hinkle 2017** | 18 *(3-39)* | MMC, Oxaliplatin | Closed | 60 or 90 | 42 | 10 *(28)* | 6 *(17)* | **30-day mortality:** NR  **Overall mortality:** 7 *(19)* |
|  |  |  |  |  |  |  |  |  |
| **Kopanakis 2018** | NR | MMC, Cisplatin, Oxaliplatin, Paclitaxel, Doxorubicin, Irinotecan | Closed | 60 or 90 | 42.5 | NR | NR | NR |
|  |  |  |  |  |  |  |  |  |
| **Macrí 2009** | 10 *(0-27)** | MMC, Cisplatin, Doxorubin | Closed | 60 or 90 | 41-43 | 5 *(28)* | 0 *(0)* | NR |
|  |  |  |  |  |  |  |  |  |
| **McQuellon 2001** | NR | MMC | NR | 120 | 40.5 | NR | NR | **30-day mortality**: 5 *(8)*  **Overall mortality:** 24 *(38)* |
|  |  |  |  |  |  |  |  |  |
| **McQuellon 2007** | NR | MMC | NR | 120 | 40 | NR | NR | **30-day mortality:** NR  **Overall mortality:** 31 *(32)* |
|  |  |  |  |  |  |  |  |  |
| **Passot 2014** | NR | MMC, Cisplatin, Oxaliplatin, Irinotecan | Closed | NR | NR | NR | 91 *(42)* | **30-day mortality:** 10 *(5)*  **Overall mortality:** 27 *(12)* |
|  |  |  |  |  |  |  |  |  |
| **Tsilimparis 2013** | 22 *(2-39)** | NR | NR | NR | NR | NR | 45 *(50)* | NR |
|  |  |  |  |  |  |  |  |  |
| **Tuttle 2013** | NR | MMC | Closed | 90 | 40 | 18 *(53)* | NR | **30-day mortality:** 0 *(0)*  **Overall mortality:** NR |

Abbreviations: 5-FU, 5-fluorouracil; CRS, cytoreductive surgery; HIPEC, hyperthermic intraperitoneal chemotherapy; MMC, mitomycin-C; NR, not reported; PCI, peritoneal cancer index (range 0-39); QoL, quality of life.

***** Outcome reported as mean (range). **^₪^** Severe postoperative morbidity defined as grade III or IV according to Clavien-Dindo classification system. **^¥^** Overall mortality during complete study period, in case of cross-sectional design mortality rate of all patients who underwent CRS with HIPEC in the included years reported.
