# Supplementary material for: Systematic Review of Factors Affecting Quality of Life After Cytoreductive Surgery with Hyperthermic Intraperitoneal Chemotherapy
Source: Ann Surg Oncol. 2020 Apr 26;27(10):3973–83. doi: 10.1245/s10434-020-08379-9 (PMC7471142; doi:10.1245/s10434-020-08379-9)
Supplement: Supplementary file 4 — Supplementary material 4 (DOCX 20 kb) [file 10434_2020_8379_MOESM4_ESM.docx]

**Supplementary Table 4. Risk of bias analysis for the included studies**

|  | **Aim** | **Consecutive patients** | **Prospective collection** | **Appropriate endpoints** | **Assessment of endpoints** | **Adequate follow-up period** | **Loss to follow-up** | **Sample size calculation** | **Adequate control group** | **Contemporary groups** | **Baseline equivalence** | **Adequate statistical analyses** | **Total** |
| --- | --- | --- | --- | --- | --- | --- | --- | --- | --- | --- | --- | --- | --- |
| **Comparative studies** | | | | | | | | | | | | | |
| **Hamilton 2016** | 2 | 1 | 0 | 2 | 1 | 1 | 0 | 0 | 2 | 2 | 1 | 2 | **14** |
| **Hinkle 2017** | 1 | 1 | 1 | 2 | 1 | 2 | 1 | 0 | 2 | 2 | 1 | 1 | **15** |
| **Non comparative studies** | | | | | | | | | | | | | |
| **Albertsmeijer 2014** | 2 | 2 | 1 | 2 | 1 | 2 | 1 | 0 | n.a. | n.a. | n.a. | n.a. | **11** |
| **Bayat 2019** | 2 | 2 | 1 | 2 | 1 | 2 | 0 | 0 | n.a. | n.a. | n.a. | n.a. | **10** |
| **Chia 2016** | 1 | 2 | 1 | 2 | 1 | 2 | 1 | 0 | n.a. | n.a. | n.a. | n.a. | **10** |
| **Dodson 2016** | 1 | 1 | 1 | 2 | 1 | 2 | 1 | 0 | n.a. | n.a. | n.a. | n.a. | **9** |
| **Hill 2011** | 1 | 2 | 1 | 2 | 1 | 2 | 1 | 0 | n.a. | n.a. | n.a. | n.a. | **10** |
| **Kopanakis 2018** | 1 | 2 | 1 | 2 | 1 | 2 | 1 | 0 | n.a. | n.a. | n.a. | n.a. | **10** |
| **Macrí 2009** | 1 | 0 | 1 | 1 | 1 | 2 | 0 | 0 | n.a. | n.a. | n.a. | n.a. | **6** |
| **McQuellon 2001** | 2 | 2 | 1 | 2 | 1 | 2 | 1 | 0 | n.a. | n.a. | n.a. | n.a. | **11** |
| **McQuellon 2007** | 1 | 2 | 1 | 2 | 1 | 2 | 1 | 0 | n.a. | n.a. | n.a. | n.a. | **10** |
| **Passot 2014** | 2 | 2 | 1 | 2 | 1 | 2 | 1 | 0 | n.a. | n.a. | n.a. | n.a. | **11** |
| **Tsilimparis 2013** | 1 | 2 | 1 | 2 | 1 | 2 | 1 | 0 | n.a. | n.a. | n.a. | n.a. | **10** |
| **Tuttle 2013** | 1 | 2 | 1 | 2 | 1 | 2 | 1 | 0 | n.a. | n.a. | n.a. | n.a. | **10** |

Assessment was according to the MINORS guidelines. The items were scored 0 (not reported), 1 (reported but inadequate), or 2 (reported and adequate). The global ideal score was 24 for comparative studies and 16 for non-comparative studies. Abbreviations: MINORS, Methodological Index for Non-Randomized Studies; n.a., not applicable.
